# Interallelic and Intergenic Incompatibilities of the *Prdm9* (*Hst1*) Gene in Mouse Hybrid Sterility

**DOI:** 10.1371/journal.pgen.1003044

**Published:** 2012-11-01

**Authors:** Petr Flachs, Ondřej Mihola, Petr Šimeček, Soňa Gregorová, John C. Schimenti, Yasuhisa Matsui, Frédéric Baudat, Bernard de Massy, Jaroslav Piálek, Jiří Forejt, Zdenek Trachtulec

**Affiliations:** 1 Department of Mouse Molecular Genetics and Center for Applied Genomics, Institute of Molecular Genetics of the Academy of Sciences of the Czech Republic, Prague, Czech Republic; 2 Center for Vertebrate Genomics, Department of Biomedical Sciences, College of Veterinary Medicine, Cornell University, Ithaca, New York, United States of America; 3 Cell Resource Center for Biomedical Research, Institute of Development, Aging, and Cancer, Tohoku University, Sendai, Japan; 4 Institut de Génétique Humaine, CNRS UPR 1142, Montpellier, France; 5 Institute of Vertebrate Biology, Academy of Sciences CR, Brno and Studenec, Czech Republic; University of Arizona, United States of America

## Abstract

The Dobzhansky-Muller model of incompatibilities explains reproductive isolation between species by incorrect epistatic interactions. Although the mechanisms of speciation are of great interest, no incompatibility has been characterized at the gene level in mammals. The *Hybrid sterility 1* gene (*Hst1*) participates in the arrest of meiosis in F_1_ males of certain strains from two *Mus musculus* subspecies, e.g., PWD from *M. m. musculus* and C57BL/6J (henceforth B6) from *M. m. domesticus*. *Hst1* has been identified as a meiotic PR-domain gene (*Prdm9*) encoding histone 3 methyltransferase in the male offspring of PWD females and B6 males, (PWD×B6)F_1_. To characterize the incompatibilities underlying hybrid sterility, we phenotyped reproductive and meiotic markers in males with altered copy numbers of *Prdm9*. A partial rescue of fertility was observed upon removal of the B6 allele of *Prdm9* from the azoospermic (PWD×B6)F_1_ hybrids, whereas removing one of the two *Prdm9* copies in PWD or B6 background had no effect on male reproduction. Incompatibility(ies) not involving *Prdm9^B6^* also acts in the (PWD×B6)F_1_ hybrids, since the correction of hybrid sterility by *Prdm9^B6^* deletion was not complete. Additions and subtractions of *Prdm9* copies, as well as allelic replacements, improved meiotic progression and fecundity also in the progeny-producing reciprocal (B6×PWD)F_1_ males. Moreover, an increased dosage of *Prdm9* and reciprocal cross enhanced fertility of other sperm-carrying male hybrids, (PWD×B6-C3H.*Prdm9*)F_1_, harboring another *Prdm9* allele of *M. m. domesticus* origin. The levels of *Prdm9* mRNA isoforms were similar in the prepubertal testes of all types of F_1_ hybrids of PWD with B6 and B6-C3H.*Prdm9* despite their different prospective fertility, but decreased to 53% after removal of *Prdm9^B6^*. Therefore, the *Prdm9^B6^* allele probably takes part in posttranscriptional dominant-negative hybrid interaction(s) absent in the parental strains.

## Author Summary

Disturbed gametogenesis in the progeny of two fertile parental forms is called hybrid sterility; it is an important part of reproductive barriers between species. The Dobzhansky-Muller model of incompatibilities explains reproductive isolation between species by incorrect interactions between genes. *Hybrid sterility 1* (*Hst1*) is one of the genes causing meiotic arrest in F_1_ male hybrids between certain *Mus musculus musculus* (e.g., the PWD strain) and *M. m. domesticus* (C57BL/6J etc.) mice. *Hst1*, the first mammalian candidate for a speciation gene, was identified as a meiotic PR/SET-domain gene, *Prdm9*, but the mechanism causing sterility has remained unknown. While the F_1_ male offspring of C57BL/6J males and PWD females produce no sperm, the males from the reciprocal cross using PWD males and C57BL/6J females yield progeny. Here we show that the meiotic progress and fertility of hybrid males from both F_1_ crosses improved by removal as well as overexpression of the C57BL/6J allele of *Prdm9*, suggesting that *Prdm9* interactions not present in the parental species (incompatibilities) play a role in hybrid sterility. Furthermore, the *Prdm9* dosage also controlled fecundity in other F_1_ hybrids, indicating that this gene is an important regulator of mouse hybrid fertility.

## Introduction

Hybrid sterility is a condition in which two fertile parental forms produce progeny with disturbed gametogenesis. In mammals and *Drosophila*, it affects spermatogenesis more often than oogenesis [1]. Hybrid sterility acts as a reproductive barrier between species [2]. Although its molecular mechanism is of great interest, only five animal genes involved in hybrid sterility have been cloned and characterized, four of them from *Drosophila* [3]–[9]. The Dobzhansky-Muller model of incompatibilities of genes [10] explains the reproductive isolation between species by their incorrect epistatic interactions. These interactions (or lack of the correct ones) result in hybrid fitness reduction, probably because the combination of the diverged alleles of the interactors did not pass through natural selection.

**Figure 1:**
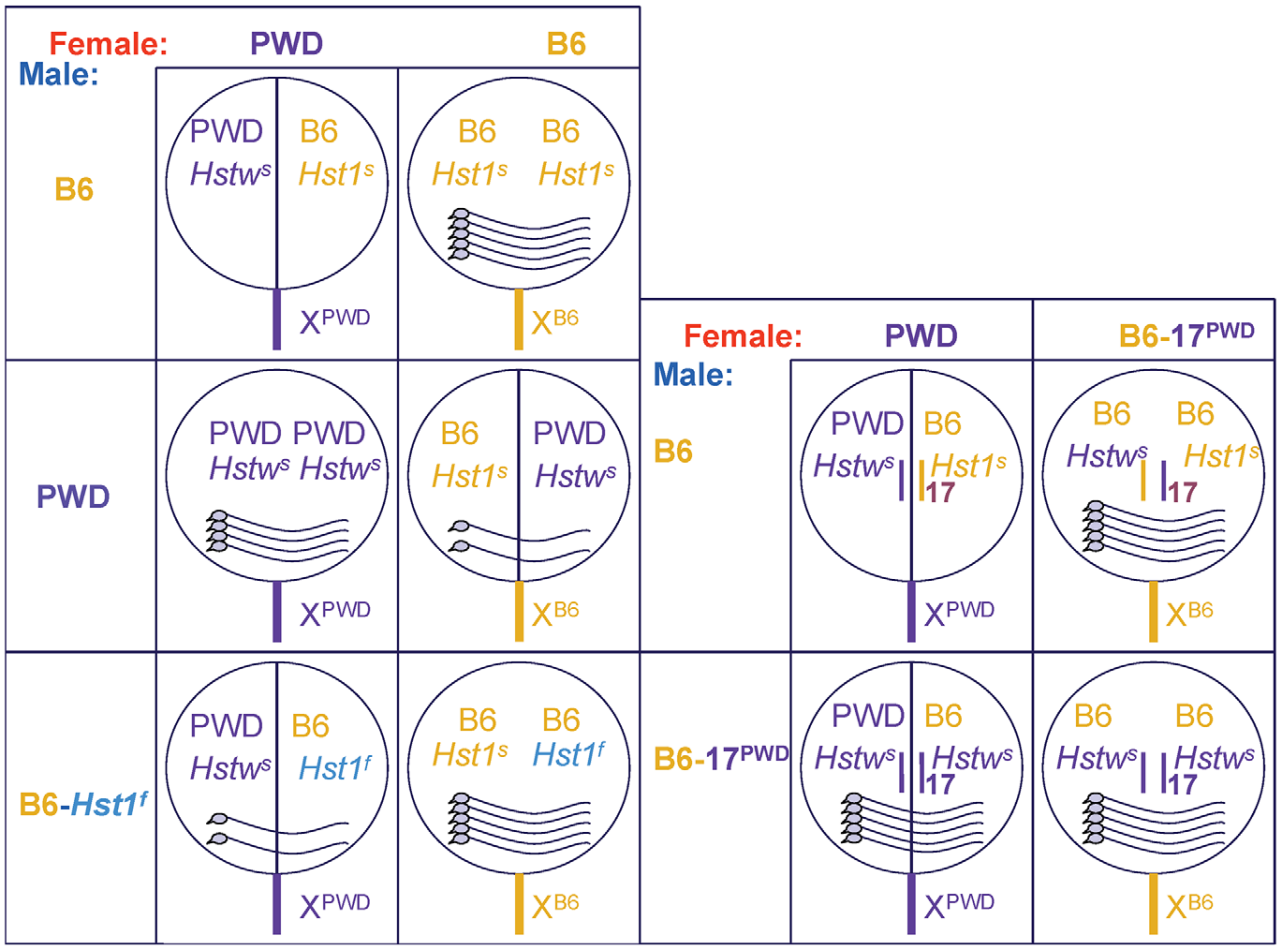
Fertility of male offspring resulting from various crosses. The female parents are shown from left to right and male parents from top to bottom. The circles symbolize genomes (chrX sticking out), intersubspecific hybrids have the circles split in halves; the pictures of sperm cells within the circles indicate the degree of fertility. Except for the (PWD×B6)F_1_ hybrids, all males carry sperm; female offspring from all indicated crosses are fertile.

The mouse *Hybrid sterility 1* gene (*Hst1*) is one of the major genes causing meiotic arrest in F_1_ male hybrids between *Mus m. musculus* (Mmm) mice harboring the *Hstw^s^* allele (e.g., the PWD strain) and laboratory strains bearing the *Hst1^s^* allele [11]. Strains with the *Hst1^s^* allele include *Mus m. domesticus* (Mmd)-derived C57BL/6J (henceforth B6) and various substrains of the 129 strain. While the male offspring from the crosses of *Hst1^s^* strains with PWD females, e.g., (PWD×B6)F_1_, are azoospermic, the males from the reciprocal crosses using PWD males [(B6×PWD)F_1_] can sire offspring (Figure 1, [6], [12]). Other laboratory strains (C3H, the congenic B6.C3H-*Hst1^f^*, etc.) harbor the *Hst1^f^* allele and produce sperm-carrying F_1_ males in crosses with PWD females [13]. The male offspring of B6 with C3H, as well as F_1_ females in all PWD, B6, and C3H crosses are fertile.

The *Hst1* gene is the first mammalian candidate for a speciation gene. *Hst1* was identified by consecutive mapping of the *Hst1* alleles [14]–[16], expression profiling [17], allelic sequencing [18], [19], and transgenic rescue [6]. The best candidate was confirmed by comparing the phenotypes of its null allele with the phenotypes of the sterile hybrids [6]. *Hst1* is also called *Prdm9* (PR-domain containing 9) or *Meisetz* (*Mei*otic gene with *SET*/PR domain and *Z*inc fingers). The product of this gene trimethylates histone 3 on lysine 4 (H3K4m3; [20]). Spermatocytes of *Prdm9^−/−^* animals and sterile hybrids arrest at the pachytene stage of meiotic prophase I, displaying defects in chromosomal pairing and sex body formation, as well as downregulation of meiotic genes. The downregulation correlates with a lower level of H3K4m3 at promoters, at least in the case of the *Morc2b* gene (*4932411A10Rik*; [6], [20]). The *Morc2b* mRNA is elevated in (PWD×B6-C3H.*Hst1^f^*)F_1_ compared to (PWD×B6)F_1_ prepubertal testis, while the levels of all known *Prdm9* transcripts are similar [6].

The chromatin of mouse meiotic recombination hotspots is marked by H3K4m3 at the start of meiosis [21]. Genetic mapping of a gene acting in *trans* to influence the activity of recombination hotspots also led to the identification of *Prdm9* [22], [23]. PRDM9 binds DNA at recombination hotspots via its zinc-fingers (ZnFs) *in vitro*, and genetic manipulation of ZnFs changes the localization of the hotspots [22], [24].

The *Prdm9* genes from C3H and B6 strains (alleles *Hst1^f^* and *Hst1^s^*, respectively), display many polymorphisms [6]. The difference that may underlie hybrid sterility is the number of C-terminal ZnFs [6]. The number of ZnFs corresponds to *Hst1* alleles in other classical Mmd laboratory strains [19], [25]. The ZnF-encoding region of the PWD allele differs from both C3H and B6 [6], but whether *Prdm9^PWD^* is identical to *Hstw^s^* and whether it contributes to hybrid sterility is not known. Although accelerated evolution of the minisatellite-like ZnF-encoding region of PRDM9 was manifested in human and animals [26], it remains to be shown whether PRDM9 has a more general role in speciation.

The *Prdm9^B6^* allele is necessary but not sufficient for hybrid sterility. The *Hstw* gene from Mmm is also located on chr17 [12]. In the sterile (PWD×B6)F_1_ males, chr17 carries the combination *Hstw^s^*/*Prdm9^B6^*, but the same genotype in the B6 background of (B6.PWD-Chr17×B6)F_1_ yields fertile males (Figure 1; [27]). It was shown recently that *Hstw^s^*/*Prdm9^B6^* is the only combination resulting in a complete meiotic arrest in (PWD×B6)F_1_ hybrid males, because both *Prdm9^B6^*/*Prdm9^B6^* and *Hstw^s^*/*Hstw^s^* homozygotes on the same background were fertile [12]. However, even on the F_1_ background, the *Hstw^s^*/*Prdm9^B6^* combination does not always lead to azoospermic males, as the reciprocal (B6×PWD)F_1_ males carry sperm. Thus, mouse hybrid sterility reflects incompatibilities among multiple hybrid sterility loci, one of them being the *Prdm9* gene [12].

Here we manipulated the dosage and allelic combinations of *Prdm9* in an attempt to characterize the role of this mouse hybrid sterility gene in the incompatibilities. If *Prdm9^B6^* has a dominant-negative effect(s), its deletion should alleviate the meiotic arrest and rescue the fertility of hybrids. Moreover, a *Prdm9*-overexpressing transgene might dilute the *Prdm9^B6^* incompatibility(ies), which should rescue fertility regardless of the *Prdm9* allelic origin. We show that the fertility of all F_1_ intersubspecific hybrids tested is proportional to the dosage of *Prdm9* regardless of its allele; the only exception is the combination of one *Prdm9^B6^* allele with one *Prdm9^PWD^* allele in either type of reciprocal hybrid that results into a more sterile phenotype than the corresponding hybrid harboring only one *Prdm9^PWD^* allele. This exception indicates an F_1_ hybrid-specific dominant-negative interaction(s) of *Prdm9^B6^*.

## Results

### Dosage-dependent, allele-independent rescue of fertility in the (PWD×B6)F_1_ hybrid

The fertility of azoospermic intersubspecific (PWD×B6)F_1_ hybrid males is rescued by *Prdm9^C3H^*-carrying BACs [6]. Moreover, six copies of the *Prdm9^C3H^* allele in a transgene (BAC24) increased reproductive fitness compared to two copies of the same allele (BAC5 transgene) in *Prdm9^PWD/B6^* (PWD×B6)F_1_ intersubspecific hybrid males ([6] and Table 1), although no effect of increased *Prdm9* dosage appeared on the intrasubspecific background (a mix of 129 and B6 genomes, henceforth B6 * 129 [6]). There are three possible explanations for the fertility rescue by the *Prdm9^C3H^* BACs: first, by allelic replacement; second, by increased dosage regardless of allelic origin; third, both. To distinguish among these hypotheses, a transgene harboring the *Prdm9^B6^* allele was utilized. The C57BL/6J-Tg(RP23-159N6)75Bdm strain carries two *Prdm9*-expressing copies of a B6 BAC transgene on B6 background [24]. In this background, the transgene has no effect on fertility (Table S1). After outcrossing the heterozygous transgenic males to PWD, non-transgenic F_1_ hybrid males were azoospermic (as expected), while their transgenic littermates had an increased testicular weight (TW) and carried sperm (Table 1). The increased copy number of the *Prdm9^B6^* allele also rescued fertility of azoospermic hybrids produced by another Mmm strain, STUS (Table S1). Thus the improvement of fertility by increased *Prdm9* dosage is not restricted to the C3H allele.

**Table 1:** The effect of *Prdm9* dosage on hybrid sterility.

Cross (female first)	*Prdm9*	n	TW	SC
PWD×B6-BACB6	PWD/B6	9	55	0.00^a^
PWD×B6-BACB6	PWD/B6+2B6	3	167*	18* a
PWD×B6-KO	PWD/B6	10	59	0.00
PWD×B6-KO	PWD/−	12	82*	0.06*
PWD×(B6-*Prdm9^C3H^*×KO)	PWD/−	10	94	0.10
PWD×(B6-*Prdm9^C3H^*×KO)	PWD/C3H	8	140*	1.06*
PWD×B6-*Prdm9^C3H^*	PWD/C3H	8	109	0.2
PWD×(KO×BAC5)	PWD/−	13	85	0.07
PWD×(KO×BAC5)	PWD/−+2C3H	15	168*	1.6*
PWD×(KO×BAC24)	PWD/−	9	77	0.03
PWD×(KO×BAC24)	PWD/−+6C3H	5	235*	3.9*

B6-BACB6, C57BL/6J-Tg(RP23-159N6)75Bdm mouse heterozygous for a *Prdm9^B6^* transgene; BAC5, transgenic strain with two copies of *Prdm9^C3H^*; BAC24, strain carrying six transgenic copies of *Prdm9^C3H^*; KO, B6-KO, heterozygote for the *Prdm9* knock-out; *Prdm9*, genotype at the *Prdm9* locus (maternal/paternal); −, null; +, transgenic *Prdm9* alleles; n, number of males; TW, mean weight of paired testicles in mg; SC, average sperm count (millions) in paired caput epididymides or

a in the left epididymis by a different method;

* significantly higher (p<0.01) compared to littermates (in the row above).

To further investigate the effect of *Prdm9* dosage on hybrid sterility, we used two null alleles of *Prdm9*, a large deletion of proximal chr17 (*Sod2^df14J^* [28]) and a knock-out of *Prdm9* that removes the first five coding exons (*Prdm9^tm1Ymat^* [20]). Males hemizygous for these alleles on (129 * B6) as well as on B6 background display similar fertility parameters as their littermates ([20], [29], and Table S2). To determine the effect of the null alleles on intersubspecific F_1_ hybrids, the hemizygous males were outcrossed to PWD. Unlike their azoospermic *Prdm9^PWD/B6^* F_1_ littermates, most of the *Prdm9^PWD/−^* intersubspecific hybrids were semisterile with TW, sperm count (SC), and offspring production (offspring per female per month, OFM) significantly higher than in the (PWD×B6)F_1_ littermates (Table 1 and Table S3). All *Prdm9^PWD/−^* hybrid males resulting from the cross of PWD females with *Prdm9^B6/−^* on B6 background carried a low but detectable amount of sperm and most of them produced offspring (0.3±0.2 OFM). As an additional control, we introduced the *Prdm9^tm1Ymat^* null allele into the PWD background. Here, one copy of *Prdm9^PWD^* was sufficient to maintain the fertility parameters of the *Prdm9^PWD/PWD^* littermates (Table S2). Therefore, the (PWD×B6)F_1_ hybrid genetic background appears to be more sensitive to low *Prdm9* dosage than that of either parent. Both addition and removal of *Prdm9^B6^* improves the phenotype of (PWD×B6)F_1_ males. The fertility rescue of azoospermic hybrid males by the *Prdm9* null alleles, albeit partial, suggests that an aberrant interaction(s) of *Prdm9^B6^* occurs in (PWD×B6)F_1_ hybrids that is not present in the parental strains.

### Incompatibility of *Prdm9^B6^* in (PWD×B6)F_1_ males

The *Prdm9^C3H^* allele rescues the fertility phenotype of the (PWD×B6)F_1_ hybrid in a dosage-dependent manner. To compare the effect of a single C3H allele and a null allele on hybrid males resulting from a cross segregating these alleles, we utilized the congenic strain B6-*Prdm9^C3H^*. After outcrossing animals hemizygous for *Prdm9* to this congenic and then crossing the preselected *Prdm9^C3H/−^* males to PWD females, the resulting *Prdm9^PWD/−^* hybrids had a significantly lower TW and SC than their *Prdm9^PWD/C3H^* littermates (Table 1). The *Prdm9^PWD/−^* hybrids produced markedly less progeny (0.3±0.2 OFM) in comparison with the *Prdm9^PWD/C3H^* hybrids (3.6±0.5 OFM, Table S4). Thus the intersubspecific F_1_ hybrid males carrying *Prdm9^PWD/−^* were not just more fertile than *Prdm9^PWD/B6^*, but they were also less fertile than *Prdm9^PWD/C3H^*.

To analyze the sensitivity of *Prdm9^PWD/−^* F_1_ background, the dosage of *Prdm9^C3H^* was increased by utilizing the BAC5 (two copies of *Prdm9^C3H^*) or the BAC24 (six copies of *Prdm9^C3H^*) transgenes. Again, the fertility parameters of the F_1_ transgenic hybrids improved with *Prdm9^C3H^* dosage (Table 1; p_SC_ = 0.006, p_TW_ = 0.008, but no significant difference in relative testes weight: 6.6 versus 7.3, p = 0.33). The *Prdm9* dosage effect is therefore observed in both *Prdm9^PWD/B6^* and *Prdm9^PWD/−^* F_1_ hybrids. In conclusion, the B6 allele displays different properties than the C3H allele when present in one copy in the *Prdm9^PWD/B6^* F_1_ hybrid male, because it decreases the fertility compared to *Prdm9^PWD/−^* F_1_ hybrid. This could be the result of a dominant-negative interaction of *Prdm9^B6^* with specific loci in (PWD×B6)F_1_ and/or with the *Prdm9^PWD^* allele.

### The semisterility of *Prdm9^PWD/−^* maintains characteristics of hybrid sterility

Hybrid sterility is most often sex-specific [1] and dependent on the origin of parents. The (PWD×B6)F_1_ hybrid displays a complete male-specific arrest of gametogenesis that is at least partially alleviated in reciprocal (B6×PWD)F_1_ and in 94% of males from the backcross ((PWD×B6)×B6) [12]. To determine whether the semisterility of the *Prdm9^PWD/−^* intersubspecific males displays the same features, additional phenotyping was performed. All four tested *Prdm9^PWD/−^* females produced offspring, in a number (4.8±0.8 OFM) similar to their *Prdm9^PWD/B6^* littermates (4.5±0.2) and (PWD×B6-*Prdm9^C3H^*)F_1_ females (5.5±0.6). The semisterility of *Prdm9^PWD/−^* F_1_ hybrids is thus male-specific. When the *Prdm9^PWD/−^* F_1_ females were backcrossed to PWD, the resulting BC_1_ *Prdm9^PWD/−^* hemizygous males displayed a four-fold increase of average SC compared to *Prdm9^PWD/−^* F_1_ hybrid males (p = 0.013), indicating that the compromised fertility is most pronounced in the F_1_ generation. The TW and SC of the reciprocal (B6-*Prdm9^−/wt^*×PWD)F_1_ *Prdm9^−/PWD^* males (Table 2) was markedly higher than that of the (PWD×B6-*Prdm9^−/wt^*)F_1_ *Prdm9^PWD/−^* hybrids (Table 1), displaying an asymmetry similar to the F_1_ cross of B6 and PWD. Thus, the fertility of *Prdm9^PWD/−^* hybrid likely suffers from an incompatibility(ies), but to a lesser degree than in the presence of *Prdm9^B6^*.

**Table 2:** Effects of *Prdm9* alleles and dosage on reciprocal hybrids.

Cross (female first)	*Prdm9*	n	BW	TW	SC
B6-KO×PWD	B6/PWD	15	25	105	0.5
B6-KO×PWD	−/PWD	11	27	174*	4.1*
(B6×B6-*Prdm9^C3H^*)×PWD	B6/PWD	7	24	97	0.3
(B6×B6-*Prdm9^C3H^*)×PWD	C3H/PWD	5	25	171*	2.2*
BAC5/BAC5×PWD	B6+2C3H/PWD	7	25	181*	3.1*
B6.PWD-Chr17×PWD	PWD/PWD^a^	7	24	220*	4.2*
B6×B6	B6/B6	3	28	195*	3.2*
PWD×PWD	PWD/PWD	4	20	119^b^	1.9*

B6-KO, heterozygote for the *Prdm9* knock-out; B6-BACB6, C57BL/6J-Tg(RP23-159N6)75Bdm male heterozygous for a *Prdm9^B6^* transgene; *Prdm9*, genotype at the *Prdm9* locus (maternal/paternal); −, null; +, transgenic *Prdm9* alleles; n, number of males; BW, body weight (g); TW, mean weight of paired testicles in mg; SC, average sperm count (millions) in paired caput epididymides;

a genotype at the entire chr17 (the rescue can also be due to a different gene, *Hstw*);

* significantly higher than in *Prdm9^B6/PWD^* reciprocal hybrids (rows 1 and/or 3);

b significantly higher relative testis weight (TW per body weight) compared to animals in rows 1 and 3 (p<0.002).

### *Prdm9* dosage and alleles affect spermatogenesis of reciprocal (B6×PWD)F_1_ hybrids

Previously, the fertility of the azoospermic (PWD×B6)F_1_ males resulting from the cross of PWD females with B6 males was rescued by *Prdm9^C3H^* overexpression [6], but *Prdm9* dosage has not been studied in the reciprocal, sperm-carrying (B6×PWD)F_1_ hybrids, although these males do not reach the reproductive fitness of fully fertile males (Table 2 and Table 3). To analyze the *Prdm9* dosage effect in the reciprocal hybrids, we crossed PWD males with females carrying a variable number of four different *Prdm9* alleles on B6 background. The fertility parameters of the *Prdm9^−/PWD^* hybrid males were superior to those of their (B6×PWD)F_1_ *Prdm9^B6/PWD^* littermates (Table 2). The parameters of the *Prdm9^B6/PWD^* transgenics carrying BAC5 were also better than those of the *Prdm9^B6/PWD^* control (Table 2). In contrast, BAC21 overlapping most of BAC5 but carrying truncated *Prdm9* [6] did not improve the fertility of *Prdm9^B6/PWD^* hybrids (Table S5). To discern whether a single copy of *Prdm9^C3H^* can improve the fertility of reciprocal hybrids, males from the cross ((B6×B6-*Prdm9^C3H^*)×PWD) were inspected. The increased fecundity of a subset of these males could be ascribed to the presence of *Prdm9^C3H^* (Table 2, p_TW_<0.001, p_SC_ = 0.003). The reciprocal *Prdm9^C3H/PWD^* F_1_ males displayed superior fertility parameters than the *Prdm9^PWD/C3H^* hybrids (Table S4; p_TW_ = 0.004, p_SC_ = 0.03, p_OFM_ = 0.04).

**Table 3:** Overview of male reproductive phenotypes.

*Prdm9*	−/−	PWD/B6	PWD/−	B6/PWD	PWD/C3H	PWD/PWD	B6/B6
Background	B6	F_1_	F_1_	F_1_	F_1_	PWD	B6
Sex body	21%	31%	67%	71%	88%	96%	99%
Diplotene	0.3%	5%	16%	17%	16%	18%	21%
Spermatids	<1%	<2%	30%	45%	45%	80%	74%
SC	0.000	0.000	0.06	0.4	0.4	1.9	3.2
TW	54	61	85	105	110	119^a^	195
OFM	0.00	0.00	0.3	3.6	3.4	6.3	6

*Prdm9*, genotype at *Prdm9* (maternal/paternal); Sex body, % pachytene spermatocytes that form a sex body; Diplotene, % diplotene of all primary spermatocytes; Spermatids, % round spermatids counted from the total of round spermatids and primary spermatocytes; SC, sperm count in paired caputs (millions); TW, testicular weight (mg); OFM, offspring per female per month;

a significantly higher relative TW (TW per body weight) than for males in columns 1 through 5. See Table S4 for more details and statistics.

To determine whether the fertility rescue of the reciprocal hybrids is limited to the *Prdm9^C3H^* allele and the PWD Mmm strain, we again used the C57BL/6J-Tg(RP23-159N6)75Bdm (carrying *Prdm9^B6^*) and STUS strains. The Mmm-derived strain STUS produces sterile (azoospermic or oligospermic) male offspring with B6 females [30], [31]. In agreement with this finding, the non-transgenic F_1_ male progeny of STUS males with B6 females heterozygous for the *Prdm9^B6^* BAC were sterile; however, the transgenic littermates were fertile (Table S1). Thus, a single copy of the *Prdm9^B6^* allele caused a decrease in fertility of F_1_ hybrids compared to the males having this allele removed, while the increased dosage of the same allele improved fertility in all F_1_ intersubspecific hybrid backgrounds tested.

The substitution of chr17^B6^ with chr17^PWD^ using the B6.PWD-Chr17 strain rescued fertility of (PWD×B6)F_1_ azoospermic hybrids, because the (PWD×B6.PWD-Chr17)F_1_ males are fertile [12]. To assess the role of chr17 heterozygosity and *Prdm9^PWD^* dosage in the semifertile reciprocal *Prdm9^B6/PWD^* hybrids, (B6.PWD-Chr17×PWD)F_1_ males were phenotyped. The replacement of chr17^B6^ with chr17^PWD^ in (B6×PWD)F_1_ hybrids led to full fertility (Table 2, p_TW_<0.001, p_SC_<0.001).

Thus, the reciprocal hybrids are sensitive to the same combinations of *Prdm9* alleles and dosage as the (PWD×B6)F_1_ hybrids, but with a shift towards higher fertility (Table 4 and Table S4). Because one copy of *Prdm9^B6^* reduced fecundity when added to *Prdm9^−/PWD^* F_1_ hybrids, the semifertility of the (B6×PWD)F_1_ hybrids appears to be affected by a dominant-negative interaction(s) of *Prdm9^B6^*.

**Table 4:** Males differing by the *Prdm9* allele, its dosage, or background divided into classes according to fertility.

Class	Sterile	Semisterile	Semifertile	Fertile	Fertile
TW (mg)	45 to 70	70 to 90	90 to 140	above 140	above 180
SC (×10^6^)	0.00	0.01 to 0.2	0.2 to 1.1	above 1.1	above 3.5
OFM	0.00	0.1 to 1	3 to 4	above 4	above 5
Background:	*Prdm9* a:	*Prdm9* a:	*Prdm9* a:	*Prdm9* a:	*Prdm9* a:
PWD×B6	PWD/B6	PWD/−		PWD/B6+2B6	PWD/PWD
PWD×B6			PWD/C3H	PWD/−+2C3H	PWD/−+6C3H
PWD×B6				PWD/B6+2C3H	PWD/B6+6C3H
B6×PWD			B6/PWD	−/PWD	PWD/PWD
B6×PWD				C3H/PWD	B6+2C3H/PWD
STUS×B6	STUS/B6			STUS/B6+2B6	
B6×STUS	B6/STUS			B6+2B6/STUS	
B6×B6	−/−				B6/−
B6×B6					B6/B6
B6×B6					PWD/B6
B6×B6					B6+2C3H/B6

TW, mean testicular weight; SC, mean sperm count in paired caput epididymides, OFM, offspring per female per month;

a genotype at *Prdm9* (maternal/paternal); −, null; +, added transgenic copies of *Prdm9*; Background: maternal×paternal background. Note that the two fertile classes display overlapping parameters.

### The overall fertility phenotypes correlate with the strength of meiotic arrest

**Figure 2:**
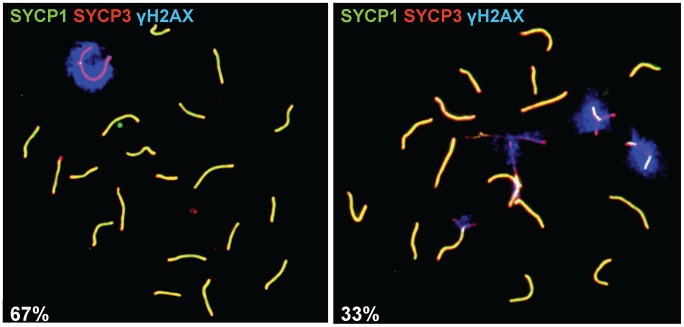
Sex body formation in the *Prdm9^PWD/−^* F_1_ hybrid male. Surface-spread nuclei of adult testicular cells treated with a hypotonic solution were indirectly labeled using antibodies marking the synaptonemal complex (anti-SYCP1 and anti-SYCP3) to discern the stage of primary spermatocytes and the phosphorylated form of the histone variant H2AX (anti-γH2AX) to visualize the sex body and then observed under a fluorescent microscope. Left, pachytene carrying a sex body (67 cases found per total of 100 nuclei from four biological replicates); right, pachytene without a sex body (33/100 nuclei).

The *Prdm9^PWD/B6^*, *Prdm9^PWD/−^*, and *Prdm9^PWD/C3H^* F_1_ hybrids show a progressive increase in overall fertility, yet even the *Prdm9^PWD/C3H^* F_1_ hybrid does not reach the parameters of other fertile males (Table 1). The fecundity defects in these hybrids could either represent different degrees of the same arrest or multiple breakdowns affecting different stages of spermatogenesis. To compare the progress of spermatogenesis in these hybrids, indirect immunofluorescence microscopy was performed on surface-spread nuclei of adult testicular cells (chromosome spreads, Table 3 and Table S4). In agreement with the SC data but in contrast to sterile hybrids carrying *Prdm9^PWD/B6^*, the chromosome spreads revealed the presence of spermatids in (PWD×B6-*Prdm9^wt/−^*)F_1_ *Prdm9^PWD/−^* males. The relative number of round spermatids in these *Prdm9^PWD/−^* testes did not reach the number observed in *Prdm9^PWD/C3H^* hybrids (Table 3 and Table S4, p<0.001). Due to an arrest at pachynema, the relative number of the four stages of primary spermatocytes in *Prdm9^PWD/B6^* was different from *Prdm9^PWD/−^* and *Prdm9^PWD/C3H^* F_1_ intersubspecific hybrids (Table 3 and Table S4, Figure S1). A sex body was formed in 67% of pachytene spermatocytes of *Prdm9^PWD/−^* hybrids (Figure 2), a higher proportion in comparison to *Prdm9^PWD/B6^* (p<0.001) but lower compared to *Prdm9^PWD/C3H^* hybrids (p = 0.001). The *Prdm9^PWD/C3H^* hybrid carried a lower ratio of pachytene spermatocytes displaying a sex body than the B6 (p = 0.004) and PWD (p = 0.03) fertile controls. The staining of spermatocyte chromosome spreads with MLH1 and SYCP1 revealed that the proportion of nuclei with fully synapsed pachytene chromosomes carrying over 20 recombination nodules is higher in the *Prdm9^PWD/−^* than in the *Prdm9^PWD/B6^* hybrids (p<0.001). The *Prdm9^PWD/−^* hybrids were similar in this respect to *Prdm9^PWD/C3H^*, but both carried less pachytene spermatocytes with completed recombination than B6 and PWD (Table S4). The meiotic phenotypes thus correlate with the TW-SC-OFM data; the *Prdm9^PWD/B6^*, *Prdm9^PWD/−^*, and *Prdm9^PWD/C3H^* F_1_ hybrids display a gradual increase in meiotic progress, yet even the *Prdm9^PWD/C3H^* F_1_ hybrid does not reach the parameters of B6 or PWD.

To determine whether the partial arrest of spermatogenesis of the semifertile reciprocal (B6×PWD)F_1_ hybrids involves meiosis I, spermatocyte chromosome spreads were analyzed. The relative number of pachytene cells carrying a sex body in the *Prdm9^B6/PWD^* hybrid was lower than in B6 and PWD (Table 3 and Table S4) and it was elevated by removing the *Prdm9^B6^* allele from the *Prdm9^B6/PWD^* hybrid (p = 0.03, Table 3 and Table S4). The number was higher in the *Prdm9^C3H/PWD^* intersubspecific male in comparison to the *Prdm9^B6/PWD^* F_1_ hybrid (p = 0.03, Table S4), and increased in the BAC5-carrying reciprocal hybrid compared to the same hybrid harboring BAC21 (p = 0.003, Table S5). Analysis of MLH1 recombination nodules indicated that *Prdm9^B6/PWD^* hybrid testes carried less pachytene spermatocytes with completed recombination than B6 or PWD (Table S4). The relative number of the four stages of primary spermatocytes in *Prdm9^B6/PWD^* differed from that in fertile males (Table S4, Figure S1). The number of offspring (OFM) correlated with meiotic phenotypes in all investigated hybrids (Table S4), thus postmeiotic incompatibilities may play a minor role in our model of F_1_ mouse hybrid sterility.

### Hybrid incompatibility(ies) of *Prdm9^B6^* is not due to a change in *Prdm9* transcript levels

**Figure 3:**
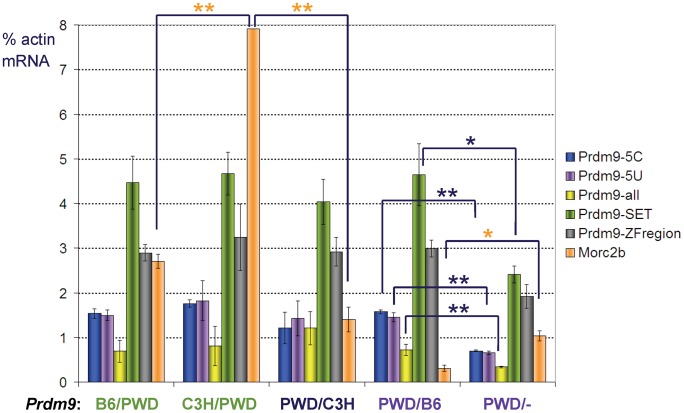
Expression of *Prdm9* and *Morc2b* in prepubertal hybrid testis. Real-time qRT-PCR was performed using RNAs of 14-day-old F_1_ intersubspecific hybrids of the indicated *Prdm9* genotypes (maternal/paternal); −, null. The animals carrying genotypes labeled by the same color were littermates. The columns indicate mRNA expression (mean ± standard deviation) relative to β-actin mRNA for five amplicons in *Prdm9* (arranged from the 5′ to 3′ of the gene) and one in *Morc2b* (orange); the asterisks mark significantly different values (*, p<0.05; **, p<0.01); the expression of *Prdm9* was similar in all except the *Prdm9^PWD/−^* F_1_ hybrids.

The fertility of the azoospermic *Prdm9^PWD/B6^* F_1_ hybrids can be rescued by *Prdm9* overexpression [6]; however, the amounts of the *Prdm9* mRNAs are similar in prepubertal *Prdm9^PWD/B6^* and *Prdm9^PWD/C3H^* hybrids that differ in prospective fertility but not in *Prdm9* dosage [6]. To understand the mechanism of the partial fertility rescue inflicted by the *Prdm9* null alleles in PWD hybrids, the transcript levels of *Prdm9* were investigated in prepubertal hybrid testes. The expression of *Prdm9* was analyzed using five qRT-PCR amplicons along the gene to account for all alternative transcripts [6]. The mRNA levels of *Prdm9* were similar in four investigated types of 14-day-old F_1_ hybrid testes carrying *Prdm9^PWD/B6^*, *Prdm9^PWD/C3H^*, *Prdm9^B6/PWD^*, and *Prdm9^C3H/PWD^*, but were significantly decreased to 52.9±2.3% in the prospectively sperm-carrying *Prdm9^PWD/−^* F_1_ hybrids compared to the prospectively azoospermic *Prdm9^PWD/B6^* littermate controls (Figure 3). In other words, the transcription from the PWD, C3H, and B6 alleles seems to be similar and dosage-dependent. Therefore, the dominant-negative interaction(s) of the *Prdm9^B6^* allele contributing to sterility in the (PWD×B6)F_1_ hybrid is most likely not a consequence of a change in the *Prdm9* transcript level.

## Discussion

Our “digital genetics“ approach (additions and subtractions of *Prdm9* copies) brings a new insight into the interactions of *Prdm9* and other hybrid sterility genes participating in the genetic Dobzhansky-Muller incompatibilities (DMIs) and controlling the reproductive fitness of intersubspecific mouse hybrids.

The phenotype of intersubspecific hybrid males is affected by *Prdm9* allelic combination and dosage (summarized in Table 4). One copy of *Prdm9^B6^* on multiple F_1_ intersubspecific hybrid backgrounds is one of the causes of reduced fertility, but a rescue can be achieved with a transgene carrying multiple copies of *Prdm9^B6^* or *Prdm9^C3H^*. The replacement of *Prdm9^B6^* with *Prdm9^C3H^* in (PWD×B6)F_1_ males significantly improves fecundity, but it nevertheless leads to semifertility that can be improved by an increased *Prdm9* dosage. The fertility rescue of hybrids by *Prdm9* transgenes is also dependent on the *Prdm9* copy number.

The F_1_ background is sensitive to *Prdm9* dosage, indicating DMIs between PWD and B6 genomes. These DMIs could involve genetic interactions between *Prdm9* and other loci, but they might also be explained by interactions between *Prdm9*-independent loci appearing as the consequence of sensitization by the *Prdm9* dosage. Although the overexpression of both *Prdm9^B6^* and *Prdm9^C3H^* alleles improved the fertility of F_1_ hybrids, the variation of effects among the *Prdm9* alleles when in one or two copies suggests either variation in the strength of the same interaction(s) or specific DMIs for each *Prdm9* allele. *Prdm9^B6^* was the only allele that resulted in a worse phenotype when one copy was added to either type of F_1_ reciprocal hybrids carrying one *Prdm9^PWD^* allele, indicating a dominant-negative effect of *Prdm9^B6^* specific for (PWD×B6)F_1_ and (B6×PWD)F_1_. The beneficial effect of the increased copy number of *Prdm9* transgenes irrespective of the transgenic allele suggests that the “toxic“ effect of the *Prdm9^B6^* incompatibility can be diluted by the overabundance of *Prdm9^B6^*.

The fertility of F_1_ hybrid males harboring chrX^B6^ was always better than that of the comparable reciprocal chrX^PWD^-carrying males, suggesting a chrX^PWD^ DMI(s) occur(s) in intersubspecific hybrids. Although theoretical options also include the interactions of chrY, mitochondrial genome or genomic imprinting, the interaction of chrX^PWD^ and chr17^PWD/B6^ was revealed by mapping hybrid sterility loci in ((PWD×B6)×B6) backcross, as well as in F_1_ using chrX subconsomics [12]. The decreased fertility of the reciprocal hybrid males *Prdm9^B6/PWD^* compared to *Prdm9^−/PWD^* and *Prdm9^C3H/PWD^* could be explained by the incompatibility of *Prdm9^B6^*-*Hstw^s^* with chrX^B6^ or autosomal loci.

Another DMI(s) not involving *Prdm9^B6^* probably also acts in F_1_ hybrids, since the null *Prdm9* alleles do not restore complete fertility. As the fertility of the reciprocal *Prdm9^−/PWD^* hybrids was superior to that of the *Prdm9^PWD/−^* F_1_ males, this DMI (or one of these DMIs) independent of *Prdm9^B6^* could involve chrX^PWD^. Both the elimination of *Prdm9^B6^* and *Prdm9* overexpression rescued fecundity in the reciprocal (B6×PWD)F_1_ hybrids, suggesting that *Prdm9^B6^* also participate(s) in a DMI(s) not involving chrX^PWD^. Supposing the same DMIs also work in the (PWD×B6)F_1_ male, a hypothesis supported by its complete sterility, there seems to be at least three sets of incompatibilities affecting the meiotic arrest in this male: *Prdm9^B6^* with chrX^PWD^, *Prdm9^B6^* with an unknown autosomal locus or loci, and chrX^PWD^ with an unknown autosomal locus or loci. Alternatively, the sets of incompatibilities could be: interautosomal B6 versus PWD sensitive to the dosage of any *Prdm9* allele; chrX^PWD^ with B6 autosomes; *Prdm9^B6^* with *Prdm9^PWD^* and/or with PWD autosomal loci. Although backcrosses using the *Prdm9* null alleles could reveal the number and map positions of the unknown autosomal loci, we already have a good candidate for one of these loci, *Hstw^s^* on chr17^PWD^.

The *Hst1^s^-Hstw^s^* (*Prdm9^B6^*-chr17^PWD^) incompatibility in F_1_ hybrids is alleviated by deletion of *Prdm9^B6^* and substitution of chr17^B6^ with chr17^PWD^ leading to increased fertility. An epistatic interaction of chr17^PWD/B6^ with chrX^PWD^ is necessary, albeit not sufficient for sterility of ((PWD×B6)×B6)BC_1_ males [12]. However, at the moment we cannot distinguish between the effects of intergenic and interallelic interactions, also because the impact of *Prdm9^PWD^* on hybrid sterility has not been directly investigated. While there is strong evidence that *Prdm9* is identical with *Hst1* in Mmd [6], we are unable to exclude that the *Hstw* locus in Mmm is linked to but different from *Prdm9*. On the other hand, *Prdm9* carries the fastest evolving ZnF domain in metazoans [26] and *Prdm9^PWD/−^* hybrids display a reduced number of pachytene spermatocytes harboring sex bodies, as well as other features of partial meiotic arrest. Therefore, the incompleteness of the rescue of hybrid fertility by *Prdm9^B6^* deletions in the (PWD×B6)F_1_ hybrid can be interpreted as the consequence of *Prdm9^PWD^* haploinsufficiency in the context of the F_1_ hybrid background, because *Prdm9^PWD/−^* F_1_ males were more affected than *Prdm9^PWD/−^* backcross males.

The hybrid sterility phenotype shows the features of spermatogenesis seen in the *Prdm9^−/−^* male and it can be alleviated by *Prdm9* transgenes [6]. *Prdm9^B6^* thus appears to participate in a loss-of-function DMI [32]. However, hybrid sterility can also be partially corrected by *Prdm9* null alleles, suggesting a gain-of-function DMI. Thus *Prdm9^B6^* might take part in multiple DMIs, both gain- and loss-of-function. Alternatively, the increased dosage of *Prdm9* could leave its part not participating in a gain-of-function DMI(s) for the normal function.

The viability of certain *Drosophila* hybrids is affected by the gene dosage of *Hmr* (*Hybrid male rescue*) [33] and by the DNA-regulatory divergence of the *Lhr* (*Lethal hybrid rescue*) gene [34]. Although the fertility of mouse hybrids can be rescued by the increased dosage of *Prdm9*, we excluded that the key difference between the *Prdm9^C3H^* and *Prdm9^B6^* alleles lies in increased transcription, because the expression of *Prdm9* mRNAs in *Prdm9^PWD/B6^*, *Prdm9^PWD/C3H^*, *Prdm9^B6/PWD^*, and *Prdm9^C3H/PWD^* prepubertal hybrid testes of the same age were similar despite the different prospective fertility (Figure 3). Increased translational efficiency remains a possibility for the key allelic difference, as *Prdm9^C3H^* and *Prdm9^B6^* differ in the 5′-untranslated region [6]. However, the polymorphism in the ZnF region of the protein products could also provide an explanation for the functional allelic difference.

As hybrid sterility can be overcome by the increased dosage of any *Prdm9* allele, one must control the number of copies in experiments designed to discern the functional sequence differences in the *Hst1* alleles. Grey et al [24] successfully used transgenesis to learn that the distribution of meiotic recombination hotspots is affected by the ZnF domain allele of *Prdm9*; no difference in the distribution was seen in the control *Prdm9^B6^* BAC transgenics. It might seem that the correction of hybrid sterility caused by the same *Prdm9^B6^* BAC could be caused by a different mechanism than redistribution of recombination hotspots. However, the increased dosage of *Prdm9* in F_1_ hybrids may overcome the DMIs and change the localization of hotspots. Nevertheless, it is unknown how a changed distribution of hotspots could lead to sterility, especially when considering that *Prdm9* function is dispensable for fertility in the dog [35], [36]. Thus (an)other function(s) of *Prdm9* may be involved in hybrid sterility, e.g., transactivation of meiotic genes.

While azoospermia was rare or absent, fertility reduced below the range found in pure species was found in one third of males in the Bavarian part of the natural house mouse hybrid zone [37]. The lack of azoospermic males can be explained by the absence of F_1_-like animals in the zone [37], [38]. F_1_ male sterility may thus be more important for establishing than for maintaining the hybrid zone. Although most of the fertility differences detected in our study were robust enough to affect the number of offspring, they are likely to have even greater impact in nature considering the sperm competition during multiple mating [39].

*Prdm9^B6^* plays a role in the complete meiotic arrest of (PWD×B6)F_1_ hybrids [6]. The importance of this finding for mouse speciation could be somewhat limited considering that only males carrying a certain allele resulting from one direction of a cross between two subspecies are affected. In this report, we demonstrated that *Prdm9* also participates in the sterility of the reciprocal (B6×STUS)F_1_ and in the partial meiotic failure of the (B6×PWD)F_1_ males. The meiosis of (PWD×B6-*Prdm9^C3H^*)F_1_ hybrids harboring another Mmd *Prdm9* allele is adversely affected by a DMI that can be alleviated by an increased *Prdm9* dosage or using the reciprocal cross, (B6-*Prdm9^C3H^*×PWD)F_1_. The reciprocal crosses of PWD and of the wild-Mmd-derived strain WSB/Ei also display differences in hybrid sterility [40]. Although many quantitative trait loci were detected in (WSB×PWD)F_2_ intercross males, heterozygosity in a region overlapping the genomic position of *Prdm9* decreases SC and relative TW; regions associated with fertility were also found on chrX^PWD^ [40]. The *Prdm9* allele of WSB is similar to C3H, being the same in the ZnF domain [23], yet WSB differs from C3H in other parts of *Prdm9* [19], [41]. Therefore, the semisterility of (PWD×WSB)F_1_ males seems to involve *Prdm9*. Admittedly, the degree of importance of *Prdm9* for mouse speciation also depends on the frequency of alleles causing reduced fertility near the hybrid zone that is currently unknown; however, the relevance of *Prdm9* for hybrid sterility now appears to be greater than shown previously.

## Materials and Methods

### Ethics statement

The mice were kept at the Specific Pathogen-Free Facility of the Institute of Molecular Genetics, Prague, and in a conventional breeding facility of the Institute of Vertebrate Biology in Studenec. Principles of laboratory animal care obeyed the Czech Republic Act for Experimental Work with Animals (Decree No. 207/2004 Sb, and the Acts Nos. 246/92 Sb, and 77/2004 Sb) fully compatible with the corresponding EU regulations and standards, namely Council Directive 806/609/EEC and Appendix A of the Council of Europe Convention ETS123.

### Mice

The STUS and PWD/Ph strains are derived from wild mice of Mmm subspecies [30], [42]. Mice carrying a deletion of chr17, *Sod2^df14J^*, were generated through embryonic stem cells [28] harboring *Prdm9^B6^* on (129×B6)F_1_ background and were transfected with BAC5. The deletion causes lethality when homozygous, it is several Mbp in length, and it includes the *Hst1* region [6]. The BAC5, BAC21, and BAC24 C3H/HeJ transgenes have no effect on fertility in non-intersubspecific hybrid males, but BAC5 and BAC24 rescue fertility of sterile hybrids [6]. The results of quantitative PCR [6] indicate that BAC24 line contains six and BAC5 two copies of *Prdm9^C3H^*; BAC21 carries two copies of truncated *Prdm9* (only the last, ZnF-encoding exon). BAC5 and BAC24 transgenes rescue fertility in *Prdm9^−/−^* (data not shown). All three transgenic lines were transferred to B6 background through 10 generations of backcrossing. The B6-*Prdm9^C3H^* (B6*B10.C3H-*Hst1^f^*) congenic carries the C3H polymorphisms at *Prdm9* and the differential segment is 3.3 Mbp (position in the mm9 genome assembly 12.5 to 15.9 Mbp) to 6.4 Mbp (10.2 to 16.5 Mbp) in length. The knock-out line *Prdm9^tm1Ymat^* was generated in 129P2/OlaHsd ES cells by replacement of the first five coding exons with LacZ [20] and maintained on mixed 129P2/OlaHsd * C57BL/6 background. The C57BL/6J-Tg(RP23-159N6)75Bdm strain (transgene Accession ID: MGI:5311012) was generated by injection of a circular BAC DNA into zygotic male pronuclei; it carries four to five copies of RP23-159N6 BAC harboring *Prdm9^B6^* (*Hst1^s^*) on B6 background, and the *Prdm9* steady-state mRNA level in primary spermatocytes is about 1.3- to 2.5-times increased compared to non-transgenic animals [24], suggesting that two *Prdm9* copies are expressed from the transgene.

### Genotyping, phenotyping, and statistics

See Text S1 for the PCR primers and conditions used for genotyping. Body weight (BW) and testicular weight (TW, from paired testicles) were determined in adult males (9 to 12- week-old). Sperm count (SC) was obtained from paired caput epididymides at room temperature [6], except for the experiments using the *Prdm9^B6^* transgene, where the entire left epididymis was extracted at 37°C [43]. Multiple biological replicates of each genotype were also analyzed for cellular phenotypes and RNA expression. Slides with surface-spread nuclei (chromosome spreads) were obtained from adult testicular cells using isotonic [44] or hypotonic [45] treatment; see Text S1 for the antibodies. Semiquantitative real-time RT-PCR was performed using total testicular RNAs of 14-day-old F1 intersubspecific hybrids exactly as described previously [6]. The significance of BW, TW, and qRT-PCR values was analyzed using Welsch's t-test, SC and OFM with Wilcoxon rank sum test, and cellular phenotypes with χ^2^ test. Unless stated otherwise, the comparison significant for TW was also significant for relative TW (TW/BW).

## Supporting Information

Figure S1 The proportions of four stages of primary spermatocytes determined by SYCP3-SYCP1-γH2AX staining of spread nuclei of adult testicular cells. −/−, *Prdm9^−/−^* on B6 background; PWD/B6, (PWD×B6)F_1_; PWD/−, hemizygous (PWD×B6-*Prdm9^B6/−^*)F_1_; B6/PWD, (B6×PWD)F_1_; PWD/C3H, (PWD×B6-*Prdm9^C3H^*)F_1_; Fert, fertile males (pooled data from B6, B6-*Prdm9^B6/−^*, and (BAC5×PWD)F_1_). Diplo, diplotene; Pach, pachytene; Zygo, zygotene; Lept, leptotene spermatocytes. The number above each column designates the total number of counted cells (average of 3.6 males per column); the asterisks indicate significant differences (χ^2^ test): *, p<0.05; **, p<0.01; ***, p<0.001.
(TIF)

Table S1 The effect of *Prdm9* dosage on hybrids of the STUS strain.
(DOC)

Table S2 The fertility of males hemizygous for the *Prdm9^m1Ymat^* knock-out.
(DOC)

Table S3 The effect of the *Sod2^df14J^* deletion and *Prdm9^tm1Ymat^* knock-out (KO) on hybrid sterility.
(DOC)

Table S4 Details of reproductive phenotypes of various males.
(DOC)

Table S5 Details of reproductive phenotypes.
(DOC)

Text S1 Supporting Materials and Methods. Antibodies, primers, PCR conditions.
(DOC)

## References

[1] Haldane JBS (1922) Sex ratio and unisexual sterility in animal hybrids. J Genetics 12: 101–109.

[2] Orr HA, Masly JP, Presgraves DC (2004) Speciation genes. Curr Opin Genet Dev 14: 675–679. 15531163 10.1016/j.gde.2004.08.009

[3] Ting CT, Tsaur SC, Wu ML, Wu CI (1998) A rapidly evolving homeobox at the site of a hybrid sterility gene. Science 282: 1501–1504. 9822383 10.1126/science.282.5393.1501

[4] Sun S, Ting CT, Wu CI (2004) The normal function of a speciation gene, *Odysseus*, and its hybrid sterility effect. Science 305: 81–83. 15232104 10.1126/science.1093904

[5] Masly JP, Jones CD, Noor MA, Locke J, Orr HA (2006) Gene transposition as a cause of hybrid sterility in *Drosophila* . Science 313: 1448–1450. 16960009 10.1126/science.1128721

[6] Mihola O, Trachtulec Z, Vlcek C, Schimenti JC, Forejt J (2009) A mouse speciation gene encodes a meiotic histone H3 methyltransferase. Science 323: 373–375. 19074312 10.1126/science.1163601

[7] Phadnis N, Orr HA (2009) A single gene causes both male sterility and segregation distortion in *Drosophila* hybrids. Science 323: 376–379. 19074311 10.1126/science.1163934 PMC2628965

[8] Bayes JJ, Malik HS (2009) Altered heterochromatin binding by a hybrid sterility protein in *Drosophila* sibling species. Science 326: 1538–1541. 19933102 10.1126/science.1181756 PMC2987944

[9] Sawamura K, Maehara K, Mashino S, Kagesawa T, Kajiwara M, et al (2010) Introgression of *Drosophila simulans* nuclear pore protein 160 in *Drosophila melanogaster* alone does not cause inviability but does cause female sterility. Genetics 186: 669–676. 20647504 10.1534/genetics.110.119867 PMC2954468

[10] Dobzhansky T (1951) Experiments on sexual isolation in *Drosophila*: X. Reproductive isolation between *Drosophila pseudoobscura* and *Drosophila persimilis* under natural and under laboratory conditions. Proc Natl Acad Sci U S A 37: 792–796. 16589029 10.1073/pnas.37.12.792 PMC1063472

[11] Forejt J, Ivanyi P (1974) Genetic studies on male sterility of hybrids between laboratory and wild mice (*Mus musculus L.*). Genet Res 24: 189–206. 4452481 10.1017/s0016672300015214

[12] Dzur-Gejdosova M, Simecek P, Gregorova S, Bhattacharyya T, Forejt J (2012) Dissecting the genetic architecture of F1 hybrid sterility in house mice. Evolution in press. doi: 10.1111/j.1558-5646.2012.01684.x. 10.1111/j.1558-5646.2012.01684.x 23106700

[13] Forejt J (1996) Hybrid sterility in the mouse. Trends Genet 12: 412–417. 8909138 10.1016/0168-9525(96)10040-8

[14] Forejt J, Vincek V, Klein J, Lehrach H, Loudova-Mickova M (1991) Genetic mapping of the *t*-complex region on mouse chromosome 17 including the *Hybrid sterility-1* gene. Mamm Genome 1: 84–91. 1799792 10.1007/BF02443783

[15] Trachtulec Z, Vincek V, Hamvas RM, Forejt J, Lehrach H, et al (1994) Physical map of mouse chromosome 17 in the region relevant for positional cloning of the *Hybrid sterility 1* gene. Genomics 23: 132–137. 7829061 10.1006/geno.1994.1468

[16] Gregorova S, Mnukova-Fajdelova M, Trachtulec Z, Capkova J, Loudova M, et al (1996) Sub-milliMorgan map of the proximal part of mouse Chromosome 17 including the *hybrid sterility 1* gene. Mamm Genome 7: 107–113. 8835526 10.1007/s003359900029

[17] Trachtulec Z, Mihola O, Vlcek C, Himmelbauer H, Paces V, et al (2005) Positional cloning of the *Hybrid sterility 1* gene: fine genetic mapping and evaluation of two candidate genes. Biol J Linn Soc 84: 637–641.

[18] Mihola O, Forejt J, Trachtulec Z (2007) Conserved alternative and antisense transcripts at the *programmed cell death 2* locus. BMC Genomics 8: 20. 17233890 10.1186/1471-2164-8-20 PMC1800895

[19] Trachtulec Z, Vlcek C, Mihola O, Gregorova S, Fotopulosova V, et al (2008) Fine haplotype structure of a chromosome 17 region in the laboratory and wild mouse. Genetics 178: 1777–1784. 18245833 10.1534/genetics.107.082404 PMC2278102

[20] Hayashi K, Yoshida K, Matsui Y (2005) A histone H3 methyltransferase controls epigenetic events required for meiotic prophase. Nature 438: 374–378. 16292313 10.1038/nature04112

[21] Buard J, Barthes P, Grey C, de Massy B (2009) Distinct histone modifications define initiation and repair of meiotic recombination in the mouse. EMBO J 28: 2616–2624. 19644444 10.1038/emboj.2009.207 PMC2738703

[22] Baudat F, Buard J, Grey C, Fledel-Alon A, Ober C, et al (2010) PRDM9 is a major determinant of meiotic recombination hotspots in humans and mice. Science 327: 836–840. 20044539 10.1126/science.1183439 PMC4295902

[23] Parvanov ED, Petkov PM, Paigen K (2010) *Prdm9* controls activation of mammalian recombination hotspots. Science 327: 835. 20044538 10.1126/science.1181495 PMC2821451

[24] Grey C, Barthes P, Chauveau-Le Friec G, Langa F, Baudat F, et al (2011) Mouse PRDM9 DNA-binding specificity determines sites of histone H3 lysine 4 trimethylation for initiation of meiotic recombination. PLoS Biol 9: e1001176 doi:10.1371/journal.pbio.1001176. 22028627 10.1371/journal.pbio.1001176 PMC3196474

[25] Forejt J, Pialek J, Trachtulec Z (2012) Hybrid male sterility genes in the mouse subspecific crosses. In: Macholan M, Baird SJE, Muclinger P and Pialek J, editors. Evolution of the House Mouse. Cambridge: Cambridge University Press, pp. 482–503.

[26] Oliver PL, Goodstadt L, Bayes JJ, Birtle Z, Roach KC, et al (2009) Accelerated evolution of the *Prdm9* speciation gene across diverse metazoan taxa. PLoS Genet 5: e1000753 doi:10.1371/journal.pgen.1000753. 19997497 10.1371/journal.pgen.1000753 PMC2779102

[27] Gregorova S, Divina P, Storchova R, Trachtulec Z, Fotopulosova V, et al (2008) Mouse consomic strains: exploiting genetic divergence between *Mus m. musculus* and *Mus m. domesticus* subspecies. Genome Res 18: 509–515. 18256238 10.1101/gr.7160508 PMC2259115

[28] Bergstrom DE, Bergstrom RA, Munroe RJ, Lee BK, Browning VL, et al (2003) Overlapping deletions spanning the proximal two-thirds of the mouse *t* complex. Mamm Genome 14: 817–829. 14724736 10.1007/s00335-003-2298-4 PMC2583125

[29] Browning VL, Bergstrom RA, Daigle S, Schimenti JC (2002) A haplolethal locus uncovered by deletions in the mouse *T* complex. Genetics 160: 675–682. 11861570 10.1093/genetics/160.2.675 PMC1461990

[30] Pialek J, Vyskocilova M, Bimova B, Havelkova D, Pialkova J, et al (2008) Development of unique house mouse resources suitable for evolutionary studies of speciation. J Hered 99: 34–44. 17965200 10.1093/jhered/esm083

[31] Vyskocilova M, Prazanova G, Pialek J (2009) Polymorphism in hybrid male sterility in wild-derived *Mus musculus musculus* strains on proximal chromosome 17. Mamm Genome 20: 83–91. 19123034 10.1007/s00335-008-9164-3

[32] Maheshwari S, Barbash DA (2011) The genetics of hybrid incompatibilities. Annu Rev Genet 45: 331–355. 21910629 10.1146/annurev-genet-110410-132514

[33] Barbash DA, Roote J, Ashburner M (2000) The *Drosophila melanogaster Hybrid male rescue* gene causes inviability in male and female species hybrids. Genetics 154: 1747–1771. 10747067 10.1093/genetics/154.4.1747 PMC1461041

[34] Maheshwari S, Barbash DA (2012) Cis-by-Trans regulatory divergence causes the asymmetric lethal effects of an ancestral hybrid incompatibility gene. PLoS Genet 8: e1002597 doi:10.1371/journal.pgen.1002597. 22457639 10.1371/journal.pgen.1002597 PMC3310770

[35] Munoz-Fuentes V, Di Rienzo A, Vila C (2011) *Prdm9*, a major determinant of meiotic recombination hotspots, is not functional in dogs and their wild relatives, wolves and coyotes. PLoS ONE 6: e25498 doi:10.1371/journal.pone.0025498. 22102853 10.1371/journal.pone.0025498 PMC3213085

[36] Axelsson E, Webster MT, Ratnakumar A, Ponting CP, Lindblad-Toh K (2012) Death of PRDM9 coincides with stabilization of the recombination landscape in the dog genome. Genome Res 22: 51–63. 22006216 10.1101/gr.124123.111 PMC3246206

[37] Turner LM, Schwahn DJ, Harr B (2011) Reduced male fertility is common but highly variable in form and severity in a natural house mouse hybrid zone. Evolution 66: 443–458. 22276540 10.1111/j.1558-5646.2011.01445.x

[38] Macholan M, Munclinger P, Sugerkova M, Dufkova P, Bimova B, et al (2007) Genetic analysis of autosomal and X-linked markers across a mouse hybrid zone. Evolution 61: 746–771. 17439609 10.1111/j.1558-5646.2007.00065.x

[39] Dean MD, Ardlie KG, Nachman MW (2006) The frequency of multiple paternity suggests that sperm competition is common in house mice (*Mus domesticus*). Mol Ecol 15: 4141–4151. 17054508 10.1111/j.1365-294X.2006.03068.x PMC2904556

[40] White MA, Steffy B, Wiltshire T, Payseur BA Genetic dissection of a key reproductive barrier between nascent species of house mice. Genetics 189: 289–304. 21750261 10.1534/genetics.111.129171 PMC3176122

[41] Keane TM, Goodstadt L, Danecek P, White MA, Wong K, et al (2011) Mouse genomic variation and its effect on phenotypes and gene regulation. Nature 477: 289–294. 21921910 10.1038/nature10413 PMC3276836

[42] Gregorova S, Forejt J (2000) PWD/Ph and PWK/Ph inbred mouse strains of *Mus m. musculus* subspecies–a valuable resource of phenotypic variations and genomic polymorphisms. Folia Biol (Praha) 46: 31–41. 10730880 10.14712/fb2000046010031

[43] Vyskocilova M, Trachtulec Z, Forejt J, Pialek J (2005) Does geography matter in hybrid sterility in house mice? Biol J Linn Soc 84: 663–674.

[44] Turner JM, Mahadevaiah SK, Fernandez-Capetillo O, Nussenzweig A, Xu X, et al (2005) Silencing of unsynapsed meiotic chromosomes in the mouse. Nat Genet 37: 41–47. 15580272 10.1038/ng1484

[45] Anderson LK, Reeves A, Webb LM, Ashley T (1999) Distribution of crossing over on mouse synaptonemal complexes using immunofluorescent localization of MLH1 protein. Genetics 151: 1569–1579. 10101178 10.1093/genetics/151.4.1569 PMC1460565

