# Supplementary figures and images for: Interallelic and Intergenic Incompatibilities of the Prdm9 (Hst1) Gene in Mouse Hybrid Sterility
Source: PLoS Genet. 2012 Nov 1;8(11):e1003044. doi: 10.1371/journal.pgen.1003044 (PMC3486856; doi:10.1371/journal.pgen.1003044)

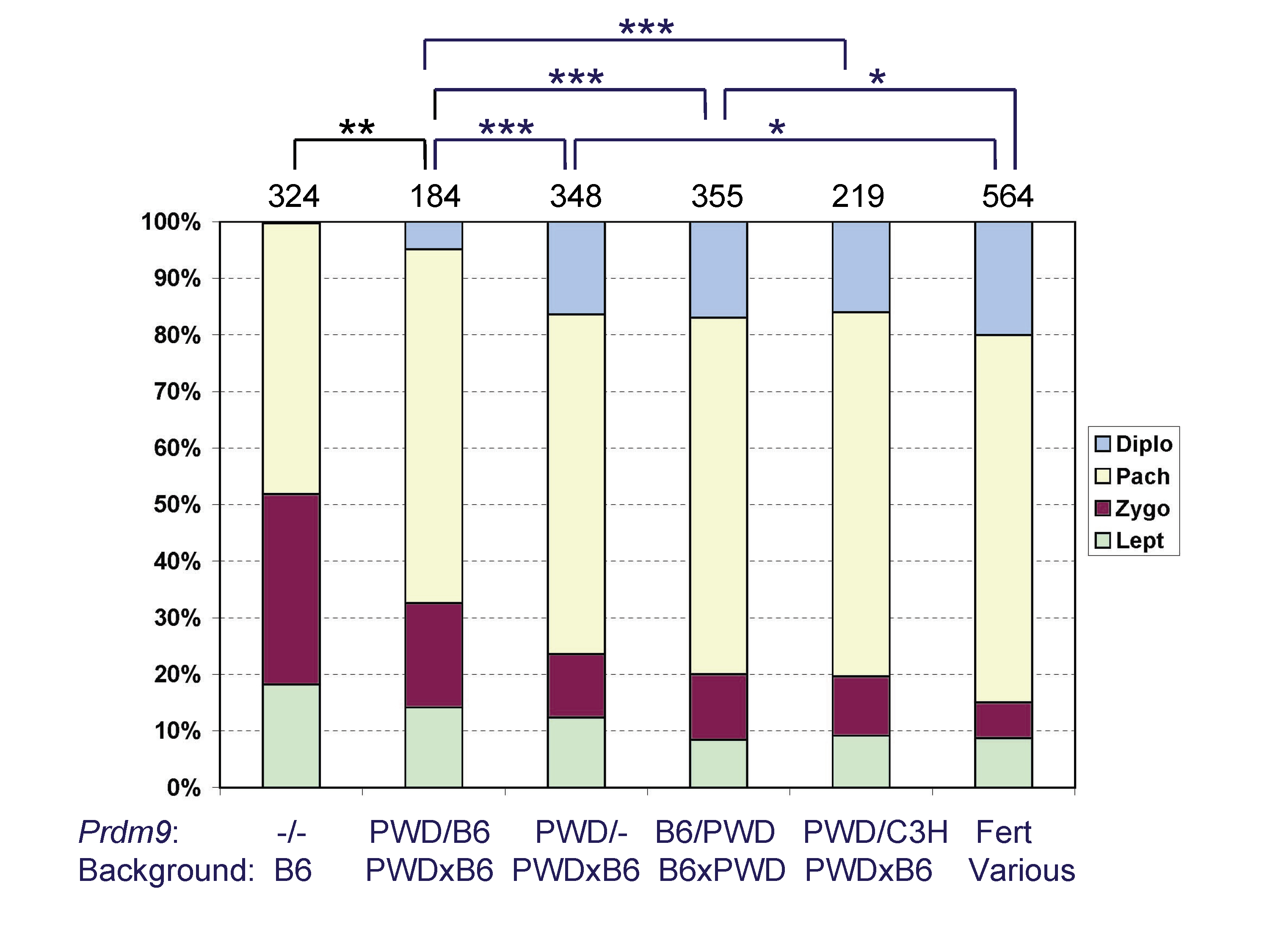

Supplement: Figure S1 — The proportions of four stages of primary spermatocytes determined by SYCP3-SYCP1-γH2AX staining of spread nuclei of adult testicular cells. −/−, Prdm9−/− on B6 background; PWD/B6, (PWD×B6)F1; PWD/−, hemizygous (PWD×B6-Prdm9B6/−)F1; B6/PWD, (B6×PWD)F1; PWD/C3H, (PWD×B6-Prdm9C3H)F1; Fert, fertile males (pooled data from B6, B6-Prdm9B6/−, and (BAC5×PWD)F1). Diplo, diplotene; Pach, pachytene; Zygo, zygotene; Lept, leptotene spermatocytes. The number above each column designates the total number of counted cells (average of 3.6 males per column); the asterisks indicate significant differences (χ2 test): *, p<0.05; **, p<0.01; ***, p<0.001. (TIF) [file pgen.1003044.s001.tif]
