# Supplementary material for: Interallelic and Intergenic Incompatibilities of the Prdm9 (Hst1) Gene in Mouse Hybrid Sterility
Source: PLoS Genet. 2012 Nov 1;8(11):e1003044. doi: 10.1371/journal.pgen.1003044 (PMC3486856; doi:10.1371/journal.pgen.1003044)
Supplement: Table S1 — The effect of Prdm9 dosage on hybrids of the STUS strain. (DOC) [file pgen.1003044.s002.doc]

**Table S1:** The effect of *Prdm9* dosage on hybrids of the STUS strain

| Cross (female first) | *Prdm9* | n | BW | TW | SC |
| --- | --- | --- | --- | --- | --- |
| STUS x B6-BACB6 | STUS/B6 | 5 | 22 | 66 | 0.00 |
| STUS x B6-BACB6 | STUS/B6+2B6 | 7 | 22 | 150* | 16* |
| B6-BACB6 x STUS | B6/STUS | 2 | 19 | 66 | 0.09 |
| B6-BACB6 x STUS | B6+2B6/STUS | 2 | 19 | 155 | 21* |
| B6-BACB6 x B6 | B6+2B6/B6 | 6 | 22 | 167* | 21* |
| B6 x B6 | B6/B6 | 8 | 23 | 172* | 22* |
| STUS x STUS | STUS/STUS | 6 | 15^a^ | 128* | 17* |

B6-BACB6, C57BL/6J-Tg(RP23-159N6)75Bdm male heterozygous for a *Prdm9^B6^* transgene; *Prdm9*, genotype at the *Prdm9* locus (maternal/paternal); +, transgenic *Prdm9* alleles; n, number of males; BW, body weight (g); TW, mean weight of paired testicles in mg; SC, average sperm count (millions) in the left epididymis; *significantly higher than in *Prdm9^STUS/B6^* hybrids (in the first row); ^a^significantly lower than all other males in this table, suggesting that the smaller TW of STUS compared to B6 is due to lower BW, not infertility; indeed STUS has a higher relative TW (TW/BW) than B6: 8.4 versus 7.6 (p=0.02).
