# Supplementary material for: Interallelic and Intergenic Incompatibilities of the Prdm9 (Hst1) Gene in Mouse Hybrid Sterility
Source: PLoS Genet. 2012 Nov 1;8(11):e1003044. doi: 10.1371/journal.pgen.1003044 (PMC3486856; doi:10.1371/journal.pgen.1003044)
Supplement: Table S2 — The fertility of males hemizygous for the Prdm9m1Ymat knock-out. (DOC) [file pgen.1003044.s003.doc]

**Table S2:** The fertility of males hemizygous for the *Prdm9^m1Ymat^* knock-out

| Genotype | Background | n | TW±SE | SC±SE |
| --- | --- | --- | --- | --- |
| *Prdm9^B6/-^* | (129 * B6) | 3 | 249±8^a^ | 4.1±0.2 |
| *Prdm9^B6/B6^* | (129 * B6) | 5 | 210±12^a^ | 3.7±0.1 |
| *Prdm9^B6/-^* | B6(BC_3_) | 5 | 197±7 | 4.4±0.5 |
| *Prdm9^B6/B6^* | B6(BC_3_) | 5 | 201±6 | 4.9±0.4 |
| *Prdm9^PWD/-^* | PWD(BC_3_) | 15 | 101±2^b^ | 2.7±0.3 |
| *Prdm9^PWD/PWD^* | PWD(BC_3_) | 8 | 111±4^b^ | 2.9±0.4 |

n, number of males; -, null; TW, weight of paired testicles (mg); SC, sperm count in paired caput epididymides (millions); SE, standard error; ^a^, ^b^, differences between littermates probably reflect body weight, as relative TW (TW/BW) is similar (^a^, p=0.21; ^b^, p=0.63).
