# Supplementary material for: Interallelic and Intergenic Incompatibilities of the Prdm9 (Hst1) Gene in Mouse Hybrid Sterility
Source: PLoS Genet. 2012 Nov 1;8(11):e1003044. doi: 10.1371/journal.pgen.1003044 (PMC3486856; doi:10.1371/journal.pgen.1003044)
Supplement: Table S3 — The effect of the Sod2df14J deletion and Prdm9tm1Ymat knock-out (KO) on hybrid sterility. (DOC) [file pgen.1003044.s004.doc]

**Table S3:** The effect of the *Sod2^df14J^* deletion and *Prdm9^tm1Ymat^* knock-out (KO) on hybrid sterility

| Cross (female first) | *Prdm9* | n | TW±SE | SC±SE | OFM±SE |
| --- | --- | --- | --- | --- | --- |
| PWD x (B6*129)/KO | PWD/B6 | 9 | 64±9 | 0.0±0.0 | n.a. |
| PWD x (B6*129)/KO | PWD/- | 13 | 106±28^a^ | 0.4±0.5^a^ | n.a. |
| PWD x (B6*129)/*Sod2^df14J^* | PWD/B6 | 13 | 62±2 | 0.0±0.0 | 0.0±0.0 |
| PWD x (B6*129)/*Sod2^df14J^* | PWD/- | 27 | 102±4^a^ | 0.5±0.2^a^ | 2.8±0.5^a^ |
| PWD x (B6*129)/*Sod2^df14J^* | PWD/-+2C3H | 11 | 124±6^b^ | 1.0±0.2^b^ | 4.3±1.1^a^ |
| PWD x (B6*129)/*Sod2^df14J^* | PWD/B6+2C3H | 27 | 159±4^c^ | 2.8±0.2^c^ | 6.5±0.3^b^ |

n, number of males; -, null; +, transgenic *Prdm9* alleles; TW, testes weight (mg); SC, sperm count in paired caput epididymides (millions); OFM, offspring per female per month (n=3); SE, standard error; n.a., not analyzed; ^a^, ^b^, ^c^ significantly higher (p<0.01) compared to: ^a^PWD/B6 littermates, ^b^PWD/- littermates, ^c^PWD/-+2C3H littermates.
