# Supplementary material for: Interallelic and Intergenic Incompatibilities of the Prdm9 (Hst1) Gene in Mouse Hybrid Sterility
Source: PLoS Genet. 2012 Nov 1;8(11):e1003044. doi: 10.1371/journal.pgen.1003044 (PMC3486856; doi:10.1371/journal.pgen.1003044)
Supplement: Table S4 — Details of reproductive phenotypes of various males. (DOC) [file pgen.1003044.s005.doc]

**Table S4:** Details of reproductive phenotypes of various males

| *Prdm9* | -/- | PWD/B6 | PWD/- | B6/PWD | PWD/C3H | -/PWD | C3H/PWD | PWD/PWD | B6/B6 |
| --- | --- | --- | --- | --- | --- | --- | --- | --- | --- |
| Background | B6 | F_1_ | F_1_ | Rec.F_1_ | F_1_ | Rec.F_1_ | Rec.F_1_ | PWD | B6 |
| Phenotype | (n) | (n) | (n) | (n) | (n) | (n) | (n) | (n) | (n) |
| Sex body | 21%(62) | 31%(55) | 67%*(100) | 71%*(69) | 88%**(154) | 91%**(90) | 96%***(132) | 96%***(101) | 99%***(90) |
| Zygotene | 34%(324) | 18%(184) | 11%(348) | 12%(355) | 11%(219) | 6%(363) | 9%(268) | 5%(684) | 6%(624) |
| Diplotene | 0.3%(324) | 5%(184) | 16%(348) | 17%(355) | 16%(219) | 23%(363) | 19%(268) | 18%(684) | 21%(624) |
| mPSCs | 0%(27)^a^ | 12%(215) | 44%*(165) | 44%*(232) | 40%*(111) | 58%*(48)^a^ | N.D. | 64%***(76) | 67%***(145) |
| Spermatids | 0%(83) | <2%(121) | 30%*(222) | 45%**(453) | 45%**(419) | 62%***(487) | N.D. | 80%***(638) | 74%***(679) |
| SC | <0.001 | <0.001 | 0.06* | 0.4** | 0.4** | 4.1*** | 2.2*** | 1.9*** | 3.2*** |
| TW (mg) | 54 | 61 | 85* | 105* | 110** | 174*** | 171*** | 119** | 195*** |
| rTW (mg/g) | 1.7 | 2.5 | 3.5* | 4.2* | 3.8* | 7.6*** | 7.0*** | 6.0*** | 7.1*** |
| Offspring/FM | 0(3) | 0(7) | 0.3*(3) | 3.6*(2) | 3.4**(9) | N.D. | 7.7***(5) | 6.3***(6) | 6**(3) |
| Fertility | Sterile | Sterile | Semisterile | Semifertile | Semifertile | Fertile | Fertile | Fertile | Fertile |

*Prdm9*, genotype at *Prdm9* (maternal/paternal); F_1_, (PWD x B6)F_1_; Rec.F_1_, (B6 x PWD)F_1_; Sex body, % pachytene spermatocytes (PSCs) that form sex body structure (Anderson method and labeling of SYCP1, SYCP3, and γH2AX); Zygotene, % zygotene of all primary spermatocytes; Diplotene, % diplotene spermatocytes (see Figure S1 for other stages); mPSCs, % pachytene spermatocytes carrying all autosomes synapsed and over 20 MLH1 foci per nucleus (of the total PSCs, Turner method, labeling SYCP1 and MLH1, confocal microscopy); Spermatids, % round spermatids counted from the total number of round spermatids and primary spermatocytes (Anderson method, labeling of SYCP3 and nuage); SC, sperm count per paired caputs (an average from multiple crosses, in millions); TW, testis weight per paired testicles (averages from multiple crosses); rTW, relative testis weight (TW/BW); n, number of cells (in top five rows) or the number of mated males in **O**ffspring per **F**emale per **M**onth (for the numbers of males utilized to determine TW and SC, see other tables); N.D., not determined; ^a^cells from a single male; *significantly different (p<0.05) from (PWD x B6)F_1_ *Prdm9^PWD/B6^*; **, significantly diverged than both *Prdm9^PWD/B6^* and (PWD x B6-KO)F_1_ *Prdm9^PWD/-^*; ***, significantly different from *Prdm9^PWD/B6^*, *Prdm9^PWD/-^*, *Prdm9^B6/PWD^*, and *Prdm9^PWD/C3H^*. The colors highlight different phenotypes.
