# Supplementary material for: Interallelic and Intergenic Incompatibilities of the Prdm9 (Hst1) Gene in Mouse Hybrid Sterility
Source: PLoS Genet. 2012 Nov 1;8(11):e1003044. doi: 10.1371/journal.pgen.1003044 (PMC3486856; doi:10.1371/journal.pgen.1003044)
Supplement: Table S5 — Details of reproductive phenotypes. (DOC) [file pgen.1003044.s006.doc]

**Table S5:** Details of reproductive phenotypes

| Cross | B6 x PWD | BAC21 x PWD | B6-C3H.*Prdm9* x PWD | BAC5 x PWD |
| --- | --- | --- | --- | --- |
| *Prdm9* | B6/PWD | B6/PWD | C3H/PWD | B6+2C3H/PWD |
| Background | (B6 x PWD)F_1_ | (B6 x PWD)F_1_ | (B6 x PWD)F_1_ | (B6 x PWD)F_1_ |
| Phenotype | (n) | (n) | (n) | (n) |
| Sex body | 71%(69) | 69%(61) | 96%*(132) | 92%*(79) |
| mPSCs | 44%(232) | 44%(39)^a^ | N.D. | 56%(36)^a^ |
| Spermatids | 45%(453) | 42%(163)^a^ | N.D. | 64%*(143)^a^ |
| SC | 0.4 | 0.1 | 2.2* | 3.6* |
| TW | 105 | 84 | 171* | 185* |
| rTW | 4.2 | 3.1 | 7.0* | 7.2* |
| Fertility | Semifertile | Semifertile | Fertile | Fertile |

Cross (female x male): BAC5, transgenic strain with two copies of *Prdm9^C3H^*; BAC21, strain carrying two truncated transgenic copies of *Prdm9^C3H^*; *Prdm9*, genotype at *Prdm9* (maternal/paternal); Sex body, % pachytene spermatocytes (PSCs) that form sex body structure (Anderson method and labeling of SYCP1, SYCP3, and γH2AX); mPSCs, % pachytene spermatocytes carrying all autosomes synapsed and over 20 MLH1 foci per nucleus (of the total PSCs, Turner method, labeling SYCP1 and MLH1, confocal microscopy); Spermatids, % round spermatids counted from the total number of round spermatids and primary spermatocytes (Anderson method, labeling of SYCP3 and nuage); SC, sperm count per paired caputs (an average from multiple crosses, in millions); TW, testis weight per paired testicles (averages from multiple crosses, mg); rTW, relative testis weight (TW/BW, in mg/g); n, number of cells (in top three rows; for the numbers of males utilized to determine TW and SC, see other tables); N.D., not determined; ^a^cells from a single biological sample per genotype processed at the same time with the control; *significantly higher (p<0.05) than (BAC21 x B6)F_1_ *Prdm9^PWD/B6^* (in the second column). The colors highlight different phenotypes.
