# Supplementary material for: Interallelic and Intergenic Incompatibilities of the Prdm9 (Hst1) Gene in Mouse Hybrid Sterility
Source: PLoS Genet. 2012 Nov 1;8(11):e1003044. doi: 10.1371/journal.pgen.1003044 (PMC3486856; doi:10.1371/journal.pgen.1003044)
Supplement: Text S1 — Supporting Materials and Methods. Antibodies, primers, PCR conditions. (DOC) [file pgen.1003044.s007.doc]

Supplemental METHODS

The following antibodies were used: primary, rabbit polyclonal anti-SCP1 (synaptonemal complex 1, SYCP1; Abcam ab15087), mouse monoclonal anti-γH2AX (anti-phospho-Histone H2A.X (Ser139), Millipore 05-636), and mouse monoclonal anti-SCP3 (SYCP3, Santa Cruz sc-74569), anti-MLH1 (MutL homolog 1, Abcam ab14206); secondary antibodies (all from Molecular Probes): goat anti-Rabbit IgG Alexa Fluor 488 (A-11034), goat anti-Mouse IgG Alexa Fluor 568 (A-11031), goat anti-Rabbit IgG Alexa Fluor 568 (A-11036), and goat anti-Mouse IgG Alexa Fluor 647 (A-21236).

PCR was performed as described previously [24]. *Prdm9* KO was tested by a three-primer PCR assay with primers (5’ to 3’): atttccctgtatcttcttcaggact (chr17:15700202-226; mm9 assembly), aggaatcttccttccttgctgtcg (chr17:15700520-497), cgccattcaggctgcgcaactgtt (LacZ). Annealing temperature was 54°C. Product length of the wild-type allele was 319 bp, of the KO allele 450 bp (KO>wt).

*Prdm9^C3H^* segregation and *Prdm9^C3H^* transgenes were genotyped for microsatellite at chr17:15588784-931 using primers TTGCTATAAAAGGACTGTTTGAT, ACACAAAGACAGAAGAAGAGGA (52°C, 144 bp B6, B6>C3H, [6]).

*Prdm9^B6^* BAC RP23-159N6 transgene was typed at the T7 end of the BAC vector (AGGAGCTGACTGGGTTGAA, CCCTATGAAGATCCTCTCCCTA, 53°C, 123 bp) .
